# Regulation and Function of Protein Kinases and Phosphatases

**DOI:** 10.4061/2011/794089

**Published:** 2011-12-13

**Authors:** Heung-Chin Cheng, Robert Z. Qi, Hemant Paudel, Hong-Jian Zhu

**Affiliations:** ^1^Department of Biochemistry & Molecular Biology, Bio21 Molecular Sciences and Biotechnology Institute, The University of Melbourne, Parkville, VIC 3010, Australia; ^2^Department of Biochemistry, Hong Kong University of Science and Technology, Clear Water Bay, Kowloon, Hong Kong, China; ^3^Bloomfield Center for Research in Aging, Lady Davis Institute for Medical Research, Sir Mortimer B. Davis-Jewish General Hospital, Montreal, QC, Canada H3T 1E2; ^4^The Department of Neurology and Neurosurgery, McGill University, Montreal, QC, Canada H3T 1E2; ^5^Department of Surgery, Royal Melbourne Hospital, The University of Melbourne, Parkville, VIC 3052, Australia

Protein kinases and phosphatases are enzymes catalysing the transfer of phosphate between their substrates. A protein kinase catalyses the transfer of *γ*-phosphate from ATP (or GTP) to its protein substrates while a protein phosphatase catalyses the transfer of the phosphate from a phosphoprotein to a water molecule. Even though both groups of enzymes are phosphotransferases, they catalyse opposing reactions to modulate the structures and functions of many cellular proteins in prokaryotic and eukaryotic cells. Among the various types of posttranslational modifications, protein phosphorylation and dephosphorylation are the most prevalent modifications regulating the structures and functions of cellular proteins in a wide spectrum of cellular processes, ranging from cell fate control to regulation of metabolism. For example, even though protein kinase genes constitute only 2% of the genomes in most eukaryotes, protein kinases phosphorylate more than 30% of the cellular proteins [1]. Owing to the significant roles of protein kinases and phosphatases in cellular regulation, this special issue focuses on their regulation, and functions. In this issue, there are two research articles and seven reviews on various topics related to the structure, regulation and functions of protein kinases and phosphatases. Together, they give the readers a glimpse of the roles played by protein kinases and phosphatases in regulating many physiological processes in both prokaryotic and eukaryotic cells. They also highlight the complexity of the regulation of protein kinases and phosphatases.

Phosphorylation regulates protein functions by inducing conformational changes or by disruption and creation of protein-protein interaction surfaces [2, 3]. Conformational changes induced by phosphorylation are highly dependent on the structural context of the phosphorylated protein. Upon phosphorylation, the phosphate group regulates the activity of the protein by creating a network of hydrogen bonds among specific amino acid residues nearby. This network of hydrogen bonds is governed by the three-dimensional structure of the phosphorylated protein and therefore is unique to each protein. The most notable example of regulation of protein function by phosphorylation-induced conformational changes is glycogen phosphorylase [4]. Glycogen phosphorylase, made up of two identical subunits, is activated upon phosphorylation of Ser-14 of each subunit by phosphorylase kinase [4]. Phosphorylation of Ser-14 in one monomer creates a network of hydrogen bonds between the phosphate group and the side chains of Arg-43 of the same monomer as well as Arg-69 of the other monomeric subunit [5]. This network induces significant intra- and intersubunit configurational changes, allowing access of the substrates to the active sites and appropriately aligning the catalytically critical residues in the active sites for catalysis of the phosphorolysis reaction.

Phosphorylation can also modulate the function of a protein by disrupting the surfaces for protein-ligand interactions without inducing any conformational changes. For example, phosphorylation of Ser-113 of the bacterial isocitrate dehydrogenase almost completely inactivates the enzyme without inducing any significant conformational changes [6, 7]. The phosphate group attached to Ser-113 simply blocks binding of the enzyme to isocitrate. Likewise, phosphorylation can also create ligand-binding surface without inducing conformational changes. For example, tyrosine phosphorylation of some cellular proteins creates the binding sites for SH2 domains and PTB domains [8, 9].

The functions of protein kinases and phosphatases are mediated by their target substrates. Understanding how protein kinases and protein phosphatases recognise their respective substrates is one of the methods used by various investigators to elucidate the physiological functions of these important enzymes. Before completion of the human genome project, most protein kinases were discovered after the discoveries of their physiological protein substrates. The most notable example is phosphorylase kinase which was discovered after glycogen phosphorylase was discovered to be regulated by phosphorylation. However, in the postgenomic era, the genes encoding protein kinases and phosphatases of an organism are known upon completion of the genome project. The challenge now is to identify their physiological protein substrates.

Protein kinases employ two types of interactions to recognize their physiological substrates in cells: (i) recognition of the consensus phosphorylation sequence in the protein substrate by the active site of the protein kinase and (ii) distal interactions between the kinase and the substrate mediated by binding of docking motif spatially separated from the phosphorylation site in the substrate and interaction motif or domain located distally from the active site of the kinase [1, 10]. These interactions contribute to the ability of protein kinases to recognize their protein substrates with exquisite specificity. Defining the structural basis of these interactions is expected to benefit identification of potential physiological substrates of protein kinases. Relevant to this, the orientated combinatorial peptide library approach developed in the 1990s and the more recently developed positional scanning peptide library approach allow rapid determination of the optimal phosphorylation sequence of many protein kinases [11, 12]. Notably, Mok et al. reported using this approach to define the optimal phosphorylation sequences of 61 out of 122 protein kinases encoded by the *Saccharomyces cerevisiae* genome [13]. Scanning the proteomes for proteins that contain motifs similar to the optimal phosphorylation sequence of a protein kinase will assist the identification of potential physiological substrates of the kinase [10]. Armed with the knowledge of many known three-dimensional structures of protein kinases with the peptide substrate bound to the active site, Brinkworth et al. designed the PREDIKIN program capable of predicting the optimal phosphorylation sequence from the primary structure of a protein serine/threonine kinase [14, 15]. Besides the peptide library approaches, researchers can also search for cellular proteins in crude cell or tissue lysates that are preferentially phosphorylated by a protein kinase *in vitro*. This method, referred to as “kinase substrate tracking and elucidation (KESTREL)” has led to the identification of potential physiological protein substrates of a number of protein kinases [16]. Finally, using specific synthetic small-molecule protein kinase inhibitors, researchers were able to perform large-scale phosphoproteomics analysis to identify physiological protein substrates of a specific protein kinase in cultured cells [2].

Substrate specificity of protein phosphatases is governed by interactions between interaction motifs or domains located distally from the phosphatase active site and distal docking motifs spatially separated from the target phosphorylation sites in protein substrates [17, 18]. Little is known about the role of the active site-phosphorylation site interactions in directing a protein phosphatase to specifically dephosphorylate its protein substrates. Using the oriented phosphopeptide library approach, several groups of researchers were able to define the optimal dephosphorylation sequences of several protein tyrosine phosphatases [19, 20], suggesting the active site-phosphorylation site interactions also play a role in dictating the substrate specificity of protein tyrosine phosphatases. Finally, the substrate-trapping mutant approach pioneered by Flint et al. in the last decade has allowed identification of physiological protein substrates of many phosphatases [21].

In this special issue, the two research articles focus on how pyruvate dehydrogenase kinase and Akt recognise their physiological substrates. The article by T. A. Hirani et al. explores how pyruvate dehydrogenase directs its recognition and phosphorylation by pyruvate dehydrogenase kinase. The article by R. S. Lee et al. reported results of their investigation that aims to decipher the regulatory mechanism governing substrate specificity of the various isoforms of Akt. The review article by A. M. Slupe et al. focuses on the structural basis governing how protein phosphatase 2A recognises its physiological substrates in cells.

It is well documented that aberrant regulation of protein kinases and phosphatases contributes to the development of diseases. For example, constitutive activation of many protein tyrosine phosphatases is known to cause cancer and neurodegenerative diseases such as Alzheimer's and Parkinson's diseases. Protein kinases and phosphatases are regulated by protein-protein interactions, binding of ligands, and reversible or irreversible covalent modifications such as phosphorylation and limited proteolysis. In this special issue, the article by I. Nakashima et al. summarizes how protein tyrosine kinases are regulated by redox reactions. C. F. Dick et al. reviewed how the activity of protein and acid phosphatases in yeast, plants, and other microorganisms is regulated by inorganic phosphate.

Among the cellular processes in which protein kinases and phosphatases are involved, this issue contains review articles detailing how protein kinases and phosphatases regulate cell cycle, mediate toll-like receptor signaling, and control of cell fate and potassium channel and intracellular calcium concentration in renal tubule epithelial cells.

In addition to protein phosphatases, acid phosphatases are involved in regulation of many biological processes such as an organism's adaptation to stress and hydrolysis of phosphorylcholine. This issue contains three review articles on the function, catalytic mechanism, and regulation of this important group of phosphatases.

*Heung-Chin Cheng* *Heung-Chin Cheng* *Robert Z. Qi* *Robert Z. Qi* *Hemant Paudel* *Hemant Paudel* *Hong-Jian Zhu* *Hong-Jian Zhu*
